# Ex Vivo Characterization of a Novel Iodine-123-Labelled Aminomethylchroman as a Potential Agonist Ligand for SPECT Imaging of Dopamine D_2/3_ Receptors

**DOI:** 10.1155/2014/507012

**Published:** 2014-12-25

**Authors:** Jan-Peter van Wieringen, Kora de Bruin, Henk M. Janssen, P. Michel Fransen, Anton G. M. Janssen, Peter A. van Doremalen, Martin C. Michel, Philip H. Elsinga, Jan Booij

**Affiliations:** ^1^Department of Nuclear Medicine, Academic Medical Center, University of Amsterdam, Meibergdreef 9, 1105 AZ Amsterdam, Netherlands; ^2^SyMO-Chem BV, Den Dolech 2, 5612 AZ Eindhoven, Netherlands; ^3^GE Healthcare, De Rondom 8, 5612 AP Eindhoven, Netherlands; ^4^Department of Pharmacology, Johannes Gutenberg University, Obere Zahlbacher Straße 67, 55101 Mainz, Germany; ^5^Department of Nuclear Medicine and Molecular Imaging, University Medical Center Groningen, University of Groningen, Hanzeplein 1, 9713 GZ Groningen, Netherlands

## Abstract

For imaging of dopamine D_2/3_ receptors, agonist tracers are favoured over antagonists because they are more sensitive to detection of dopamine release and because they may selectively label the high-affinity receptor state. We have developed novel D_2/3_ receptor selective agonists that can be radiolabelled with [^123^I], which label is advantageous over most other labels, such as carbon-11, as it has a longer half-life. Particularly, we considered (R) N-[7-hydroxychroman-2-yl]-methyl 4-iodobenzyl amine (compound **1**) as an attractive candidate for development as it shows high binding affinity to D_2/3_ receptors in vitro, and here we report on the characterization of this first [^123^I]-labelled D_2/3_ receptor agonist radiopharmaceutical intended for SPECT imaging. The appropriate tin precursor for [^123^I]-**1** was developed and was successfully radiolabelled with iodine-123 giving a moderate yield (30–35%) and a good purity (>95%) for [^123^I]-**1**. In biodistribution experiments in Wistar rats intravenous injection of [^123^I]-**1** resulted in a fast brain uptake, where the observed binding in the D_2/3_ receptor-rich striatum was slightly higher than that in the cerebellum 30 min to 4 h p.i. Storage phosphor imaging experiments, however, did not show specific D_2/3_ receptor binding. In conclusion, despite promising in vitro data for **1**, neither specific ex vivo binding nor high signal-to-noise ratios were found in rodents for [^123^I]-**1**.

## 1. Introduction

A disturbed dopamine system plays a role in the etiology of several neuropsychiatric disorders, including Parkinson's disease (PD) [1], schizophrenia [2], and drug addiction [3]. The prevalence of PD and schizophrenia is about 1% [4], while the prevalence of addiction (including alcoholism) is much higher [5], leading to a large disease burden.

Dopamine receptors are part of the superfamily of G-protein coupled receptors (GPCRs) and can, based on their action on adenylyl cyclase (AC), be divided into 2 subfamilies. After activation, the dopamine D_1_-like (D_1_ and D_5_) receptors stimulate AC to produce the second messenger cyclic adenosine monophosphate (cAMP) while the D_2_-like (D_2_, D_3_, and D_4_) receptors inhibit this enzyme [6, 7]. Like other GPCRs they demonstrate interconvertible high- and low-affinity states for agonists in vitro [8–11]. The D_2/3_ high-affinity state represents the active form of the receptor [12]. Changes in the density of D_2/3_ receptors in this high-affinity state seem to be more important for the pathophysiology of neuropsychiatric disorders than those of the total receptor density; in several animal models increases in striatal D_2/3_ high-affinity receptors of up to 9-fold compared to control animals were found while the total receptor number did not increase or even showed a small decrease [13].

Because agonists are selective for the high-affinity state they can distinguish a shift in receptors from low- to high-affinity states. Antagonists fail to do this because they bind with equal affinity to both the low- and high-affinity states of a receptor [14]. Several compounds of different chemical classes have been synthesized and characterized as agonist positron emission tomography (PET) radioligands (for review see [15]), which offers the potential to image the D_2/3_ high-affinity receptor in the human brain. Of them only the aporphines [^11^C]NPA (N-propylnorapomorphine), [^11^C]MNPA (2-methoxy-N-propylnorapomorphine), and the naphthoxazine [^11^C]PHNO (4-propyl-9-hydroxynaphthoxazine) are currently being evaluated in man [16–18]. [^11^C]PHNO and [^11^C]NPA showed a better sensitivity to detection of dopamine release than antagonist radiopharmaceuticals in human brain [19, 20] which confirmed earlier findings of animal studies [21, 22].

**Scheme 1:**
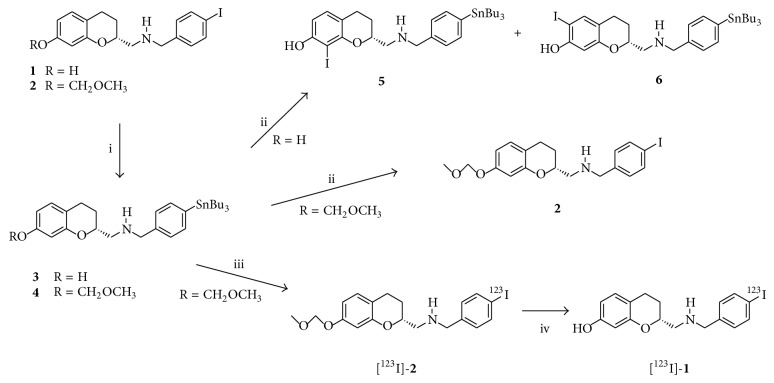
Compilation of the applied chemical conversions. Preparative synthesis includes the following: (i) hexa-n-butylditin, Pd(PPh_3_)_4_, and dioxane, 100°C. “Cold” labelling tests include the following: (ii) NaI, H_2_O_2_, and acetic acid/acetate buffer, rt. Radiolabelling includes the following: (iii) [^123^I]-NaI, H_2_O_2_, and HOAc/NH_4_OAc buffer and (iv) H_2_SO_4_, ethanol, and heat.

As of yet only [^11^C]-labelled agonist dopamine D_2/3_ receptor PET ligands have been developed successfully in humans. But because of the short half-life of [^11^C] (20.4 min), these radiopharmaceuticals have the disadvantage that they can only be used when a cyclotron is on-site or nearby. This expensive device is available in only the minority of hospitals. Interestingly, a [^18^F]-labelled aporphine ([^18^F]MCL-524) has recently been evaluated successfully in monkeys [23]. Compared to [^11^C]-labelled tracers, this tracer offers the advantage of a longer half-life (109.8 min), but studies in humans have not yet been reported, and no agonist SPECT tracer for imaging dopamine D_2/3_ receptors has been developed yet. Consequently, it is rational to develop [^123^I]-labelled (half-life 13.2 h) agonists dopamine D_2/3_ tracers for single photon emission computed tomography (SPECT) imaging because [^123^I] coupled ligands are better available because they can be distributed from the site where they are synthesized. SPECT cameras are available in the vast majority of hospitals, and because iodine-123 has a substantial half-life, studies with [^123^I]-labelled ligands can also be better scheduled than studies using [^11^C]-labelled tracers. Therefore we have synthesized and explored a novel series of potential dopamine agonists [24] that are suitable for PET or SPECT imaging. More specifically, we have developed a series of aminomethylchromans (AMCs), a class of molecules first introduced by Mewshaw et al. [25], and have found that most of these AMCs were agonists at D_2/3_ receptors and showed high-affinity and selectivity for these versus other dopamine receptor subtypes. Additionally, some of the presented compounds were successfully labelled with fluorine-18 and were subsequently used in small-animal studies [24]. These findings suggested that a particular synthesized iodide AMC-compound, compound** 1 **(see Scheme 1), is an attractive candidate for labelling with iodine-123, as this compound** 1** shows a high-affinity and selectivity for D_2/3_ receptors in their high-affinity state, a proper lipophilicity, and a high degree of agonism (see Section 2 for details). Furthermore, structure** 1** is an aryl-iodide, so it should permit the synthesis of a trialkyltin precursor that is suitable as a substrate for oxidative electrophilic radioiodination. In this study, we present the ex vivo characterization of compound** 1**. After labelling with iodine-123, classic biodistribution studies as well as storage phosphor imaging studies were performed in rats to test the suitability of this novel tracer for SPECT imaging.

## 2. Materials and Methods

Reagents, chemicals, materials, and solvents were obtained from commercial sources and were used as received: Biosolve, Merck for solvents, Cambridge Isotope Laboratories for deuterated solvents, and Aldrich, Acros, ABCR, Merck, or Fluka for chemicals, materials, and reagents. All solvents were of analytical grade (AR) quality. Moisture or oxygen-sensitive reactions were performed under an atmosphere of dry N_2_ or argon. The syntheses of compound** 1** and its MOM (CH_3_–O–CH_2_–)-protected derivative (compound** 2**) (see Scheme 1) have been reported previously [24]. Analytical thin layer chromatography (TLC) was performed on Kiesel gel F-254 precoated silica plates. Normal phase column chromatography was carried out on flash silica gel (40–63 *μ*m mesh) or regular silica gel (60–200 *μ*m), both acquired from Screening Devices B.V.

^1^H-NMR and ^13^C-NMR spectra were recorded on Varian Mercury (400 MHz for ^1^H-NMR, 100 MHz for ^13^C-NMR) spectrometers at 298 K. Chemical shifts are reported in ppm downfield from tetramethylsilane (TMS) for ^1^H NMR and applying deuterated chloroform (CDCl_3_) or other deuterated solvents as internal reference for ^13^C NMR.

HPLC-PDA/MS analyses, as used for the characterization of precursors** 3** and** 4** as well as in the labeling tests, were performed on a Shimadzu LC-10 AD VP series LC coupled to a photodiode array (PDA) detector (Finnigan Surveyor PDA Plus Detector, Thermo Electron Corporation) and an ion-trap detector (LCQ Fleet, Thermo Scientific). Analyses were executed at 298 K using an Alltech Alltima HP C18 3 *μ* column using an injection volume of 1–4 *μ*L, a flow rate of 0.2 mL min^−1^, and a MeCN in H_2_O gradient (2-minute isocratic conditions at 5% MeCN, followed by a 10-minute gradient to 100% MeCN, where both MeCN and H_2_O contain 0.1% formic acid).

[^123^I]-NaI was produced using the cyclotron at the Eindhoven site of GE Healthcare, Netherlands, and was isolated in a 0.05 M NaOH aqueous solution. Other reagents, chemicals, materials, and solvents were obtained from commercial sources and were used as received. For the separation and isolation of [^123^I]-**1** reversed phase chromatography was performed on a Waters Spherisorb S5ODS2 (150 × 4.6 mm) applying a Varian Bond Elut C18 (1 mL 100 mg) cartridge. This HPLC system was also used to assess the yield and radiochemical purity of the end product, applying a NaI-scintillation detector.

### 2.1. In Vitro Binding Data and Physiochemical Characteristics on Compound **1**

We have previously presented the in vitro data of a new series of AMCs, including those for the unlabelled compound** 1**, that is, (R) N-[7-hydroxychroman-2-yl]-methyl 4-iodobenzyl amine [24]. See Scheme 1 for the molecular structure of** 1**. Briefly, compound** 1** showed a high in vitro affinity (mean Ki of 3.79 nM and 51.8 nM for D_2_ high and D_2_ low, resp.) and selectivity for the D_2/3_  receptor_high_ over D_1_-like receptors (mean Ki for D_1_ receptor of 8.67 *μ*M). Also, the compound showed agonism for the D_2_ receptor (pEC50: cAMP: 9.2 ± 0.4, Emax: 86% ± 4.9 (% of dopamine)) and a calculated lipophilicity of log⁡D_7.4_ = 2.43 (using ClogP software). For all details with respect to the performed experiments and assays on compound** 1**, the reader is referred to our earlier work [24], wherein compound** 1** is numbered as compound** 11a**.

### 2.2. Synthetic Procedures

#### 2.2.1. (R) N-[7-Hydroxychroman-2-yl]-methyl 4-(Tri-n-butyltin)-benzyl Amine (Precursor **3**)

(R) N-[7-Hydroxychroman-2-yl]-methyl 4-iodobenzyl amine (**1**) (50 mg, 0.127 mmol), palladium tetrakis (7.3 mg, 6.3 micromol, 0.05 moleqs), hexa-n-butylditin (147 mg, 0.253 mmol, 2 moleqs), and dioxane (1 mL) were stirred at 100°C for 16 hours while the reaction mixture was kept under an argon atmosphere. According to HPLC-MS analysis no iodocompound** 1** was present any more. The mixture was cooled down to room temperature and was filtered over Celite and the filtrate was evaporated. Dissolution in CHCl_3_ was followed by washing of the organic layer with a 5% KF solution in water to remove the Bu_3_SnI byproduct. The CHCl_3_ solution was evaporated to dryness and the crude product was purified by silica column chromatography, eluting first with CHCl_3_/TEA (99/1) and then with CHCl_3_/TEA/MeOH (98/1/1). Yield: 27 mg (38%).

^1^H NMR (400 MHz, CDCl_3_, *δ*): 7.45 (d,* J* = 7.8 Hz, Ar H, 2H), 7.35 (d,* J* = 7.8 Hz, Ar H, 2H), 6.8 (d,* J* = 8.2 Hz, Ar H, 1H), 6.35 (m, Ar H, 2H), 4.05 (m, CHO, 1H), 3.95 (d,* J* = 13.3 Hz, NC*H*′H′′Ph, 1H), 3.85 (d,* J* = 13.3 Hz, NCH′*H*′′Ph, 1H), 2.85 (m, CHN, 1H), 2.75 (m, CHN, 1H), 2.7-2.6 (m, CH, 2H), 1.85 (m, CH, 1H), 1.65 (m, CH, 1H), 1.55 (m, CH_2_, 6H), 1.3 (m, CH_2_, 6H), 1.05 (m, CH_2_, 6H), 0.9 (t, CH_3_,* J* = 7.2 Hz, 9H). ^13^C NMR (100 MHz, CDCl_3_, *δ*): 156.3, 154.5, 141.2 (^1^ *J*_CSn_ = 384, 367 Hz), 137.7 (^4^ *J*_CSn_ = 10 Hz), 136.8 (^2^ *J*_CSn_ = 31 Hz), 130.0, 128.2 (^3^ *J*_CSn_ = 41 Hz), 112.5, 109.2, 103.4, 74.2, 54.1, 53.7, 29.1 (^3^ *J*_CSn_ = 20 Hz), 27.4 (^2^ *J*_CSn_ = 57 Hz), 26.3, 23.7, 13.7, 9.6 (^1^ *J*_CSn_ = 340, 325 Hz). According to HPLC-PDA/MS, compound** 3** was pure as in the chromatogram the product peak was dominant (>95%). HPLC-MS:* m/z* = 560.3 [M+H]^+^, (calcd 558.38 for C_29_H_45_NO_2_Sn).

#### 2.2.2. (R) N-[7-(Methoxymethoxy)chroman-2-yl]methyl 4-(Tri-n-butyltin)-benzyl Amine (Precursor **4**)

(R) N-[7-(Methoxymethoxy)chroman-2-yl]-methyl 4-iodobenzyl amine (**2**) (90 mg, 0.205 mmol), palladium tetrakis (11.8 mg, 10.2 micromol, 0.05 moleqs), and hexabutylditin (237 mg, 0.409 mmol, 2 moleqs) were stirred in dioxane (1 mL) at 100°C for 4 hours, while the mixture was kept under an argon atmosphere. According to HPLC-MS no iodocompound** 2** was present any more. The mixture was cooled down to room temperature and filtered over Celite. The filtrate was evaporated, and the crude product was dissolved in CHCl_3_. Washing with a 5% KF solution in water was performed so as to remove Bu_3_SnI byproduct. Finally, the CHCl_3_ layer was concentrated and the residue was purified by silica column chromatography eluting with CHCl_3_/TEA (99/1). Yield: 50 mg (41%).

^1^H NMR (400 MHz, CDCl_3_, *δ*): 7.45 (d,* J* = 7.9 Hz, Ar H, 2H), 7.35 (d,* J* = 7.9 Hz, Ar H, 2H), 6.9 (d,* J* = 8.2 Hz, Ar H, 1H), 6.53 (m, Ar H, 2H), 5.1 (s, OCH_2_O, 2H), 4.15 (m, CHO, 1H), 3.8 (s, NCH_2_Ph, 2H), 3.45 (s, OCH_3_, 3H), 2.9–2.6 (m, CH, 4H), 1.95 (m, CH, 1H), 1.75 (m, CH and NH, 2H), 1.55 (m, CH_2_, 6H), 1.3 (m, CH_2_, 6H), 1.05 (m, CH_2_, 6H), 0.9 (t,* J* = 7.3 Hz, CH_3_, 9H). ^13^C NMR (100 MHz, CDCl_3_, *δ*): 156.4, 155.3, 140.2 (^1^ *J*_CSn_ = 390, 372 Hz), 139.9 (^4^ *J*_CSn_ = 10 Hz), 136.6 (^2^ *J*_CSn_ = 31 Hz), 129.9, 127.7 (^3^ *J*_CSn_ = 41 Hz), 115.5, 108.9, 104.4, 94.5, 75.4, 55.9, 54.0, 53.7, 29.1 (^3^ *J*_CSn_ = 21 Hz), 27.4 (^2^ *J*_CSn_ = 57 Hz), 25.6, 24.1, 13.7, 9.6 (^1^ *J*_CSn_ = 340, 325 Hz). According to HPLC-PDA/MS, compound** 4** was pure as in the chromatogram the product peak was dominant (>95%). HPLC-MS:* m/z* = 604.3 [M+H]^+^, (calcd 603.3 for C_31_H_49_NO_3_Sn).

### 2.3. Labelling Tests

#### 2.3.1. “Cold” Iodination of the Unprotected Phenol Precursor **3**

Tri-n-butyltin precursor** 3** (1 mg, 1.79 *μ*mol, 12.3 moleqs) was dissolved in ethanol (1 mL) and an acetic acid buffer solution (10 mL of a pH = 4 buffer prepared by mixing 3.4 g NH_4_OAc and 8.2 g glacial acetic acid in 1 L of water). Next, NaI was added (721 microliters of a stock solution of 30 mg NaI/L 0.05 M NaOH; 0.145 *μ*mol NaI), and finally a 30% solution of H_2_O_2_ in water (1 mL) was added. After 1 hour of stirring at room temperature, the reaction mixture was analyzed by HPLC-MS. Besides the starting material** 3** that was observed most abundantly, two main products were found where these two compounds had the same molecular weight of 684.3 and showed almost identical retention times. Given this molecular weight, we attribute these two compounds to molecules** 5** and** 6**; see Scheme 1, where iodination has taken place at the (activated) ortho positions relative to the phenol hydroxy group. Furthermore, destannylated product, that is, (R) N-[7-hydroxychroman-2-yl]-methyl benzyl amine, was traced, while the desired labelled material** 1** was hardly traced at all.

#### 2.3.2. “Cold” Iodination of the MOM-Protected Precursor **4**

Tri-n-butyltin precursor** 4** (2 mg, 3.32 *μ*mol, 11.4 moleqs) was dissolved in ethanol (2 mL) and an acetic acid buffer (20 mL of a buffer solution prepared from 3.4 g NH_4_OAc and 8.2 g glacial acetic acid in 1 L of water). Next, NaI was added (1442 *μ*L of a solution prepared from 30 mg of NaI in 1 L 0.05 M NaOH solution in water; 0.29 *μ*mol NaI), and finally a 30% H_2_O_2_ solution in water (2 mL) was added. The reaction was left to stir for 1 hour at room temperature. According to HPLC-MS analysis, the starting compound** 4** was mostly present, as well as the desired iodinated MOM-protected product** 2**. No other products other than** 2** were traced, particularly not those that indicate ortho iodination, that is, products with a molecular weight of 728.3. Even after addition of another portion of NaI (0.4 mg, 2.66 *μ*mol, 0.8 moleqs) in 1 mL of water, and after further stirring of the reaction mixture for 3 hours, products other than** 2** were not traced.

### 2.4. Radiolabelling

#### 2.4.1. The Radiosynthesis of [^123^I]-**1**

Precursor compound** 4** was dissolved in ethanol at a concentration of 1 mg/mL. Typically, 50 to 80 *μ*L of this solution (50–80 *μ*g** 4**) and 100–300 *μ*L of a 0.05 M NaOH solution containing the required amount of [^123^I]-NaI (1.48–5.18 GBq/40–140 mCi at the time of handling (TOH); specific activity 185 MBq/nmol (5000 Ci/mmol at reference time)) were mixed in about 0.5 mL of a pH = 4 NH_4_OAc/HOAc buffer solution (HOAc 0.13 M and NH_4_OAc 0.044 M; prepared by adding 1.7 gr NH_4_OAc and 4 mL of glacial acetic acid to 500 mL of water). To this buffered solution, 50 *μ*L of a 30 v/v% H_2_O_2_ aqueous solution and finally 50 *μ*L of a 25 v/v% sulfuric acid aqueous solution were added. After 5 to 10 minutes the reaction mixture was passed through a C18 SPE cartridge. Rinsing with water to remove H_2_O_2_ and salts was followed by recovery of the reaction product [^123^I]-**2** in 1 mL of ethanol. Next, a small volume of a 25 v/v% sulfuric acid aqueous solution (250–300 *μ*L) was added and the mixture was heated in a stream of hot air for 10 min to achieve >95% deprotection of the MOM group. A second C18 SPE cartridge was used to isolate the deprotected product [^123^I]-**1**. Since in the MOM-deprotection step radioiodinated side products appeared, the crude product was purified using a HPLC separation step applying isocratic conditions (eluent: ethanol/0.2 M NaOAc = 65/35 (v/v)). The purified product was isolated from the HPLC eluent by using a third C18 SPE cartridge, again collecting the product in ethanol. Finally, the ethanol solvent was removed by evaporation and the product was redissolved in an isotonic pH = 4.8 NaOAc buffer containing 8–10 v/v% ethanol. Typically, the overall yield of the process was 30–35% with a final radiochemical purity exceeding 95%. The radiochemical purity and identity were determined using HPLC (the same system as used for purification); the yield was determined using an ionization chamber.

### 2.5. Animal Studies

The performed experiments are in agreement with The Dutch Experiments on Animals Act (1977) and were approved by the Animal Ethics Committee (AMC, Amsterdam, Netherlands).

#### 2.5.1. Biodistribution Studies in Rats

[^123^I]-**1** (SA ≥ 37 MBq/nmol) was received after radiolabelling in an isotonic acetate (9% EtOH) buffer (pH 4.8). The solution was passed through a membrane filter (0.2 *μ*m) and was diluted to the proper concentrations. Male Wistar rats (approximately 250 g body weight, obtained from Harlan/Charles River, Zeist, Netherlands) received an intravenous injection in the tail vein of approximately 3.7 MBq/0.4 mL buffer of [^123^I]-**1** under O_2_/CO_2_ anaesthesia. Distribution to the tissue was measured 15 min, 30 min, and 1, 2, 3, 4, and 24 h after the injection to determine the time course of uptake in several organs, including the brain. Four rats were killed at each time point via bleeding by heart puncture (to minimize contribution of blood activity) under O_2_/CO_2_ anaesthesia followed by cervical dislocation. Brain regions (cerebellum, frontal and prefrontal cortex, olfactory bulbs, pituitary, medulla with pons, midbrain, striatum, hypothalamus, hippocampus, and thalamus) and various peripheral tissues (heart, lung, liver, spleen, kidney, muscle, fat, and thymus) were quickly excised and weighted. The activity of [^123^I] was assayed in a gamma counter, and data was corrected for decay and the amount of radioactivity was presented as percentage of the injected dose, multiplied by the body weight in kilograms, per gram tissue or blood [26]. Selective uptake in the striatum was determined by calculating the ratio of radioactivity in the striatum in relation to that in the cerebellum, since the density of dopamine D_2/3_ receptor is negligible in this brain area in rats [27].

#### 2.5.2. Blocking Studies Using Storage Phosphor Imaging

To determine whether [^123^I]-**1 **binds selectively to D_2/3_ receptors in vivo, in the following experiments 5 rats received an i.v. injection of the dopamine receptor blocker haloperidol (1 mg/kg) 5 min prior to the injection with [^123^I]-**1 **(approximately 37 MBq). A control group (*n* = 5) was injected with 0.4 mL buffer 5 min prior to the injection with the radiopharmaceutical. Two hours after the injection of [^123^I]-**1 **(this time point was based on the results of the classic biodistribution study; see Results) the rats were sacrificed by bleeding through heart puncture under O_2_/CO_2_ anaesthesia. The brains were quickly removed and immediately frozen on dry ice. Then the frozen brain was sliced horizontally in 50 *μ*m thick slices. Storage phosphor imaging was done as previously described [28]. In contrast to previous studies, we used a Typhoon FLA 7000 Phosphor imager (GE Healthcare Life Sciences, Uppsala, Sweden) and Hypercassette Amersham Biosciences imaging plates which were scanned at 25 *μ*m pixel size. In this blocking experiment, storage phosphor imaging was used because this offers the opportunity to look into binding in ventral as well as dorsal parts of the striatum. In ventral parts, the expression of dopamine D_3_ receptors is higher than in more dorsal parts [19].

## 3. Results

### 3.1. Chemistry and Radiochemistry

In Scheme 1 all chemical transformations relevant to this research work are compiled. For preparing the radiolabelled AMC tracer [^123^I]-**1** we have considered and prepared the tri-n-butyl-tin precursors** 3** and** 4**, where** 3** is the phenol precursor and** 4** is the MOM-protected precursor. These two molecules have been synthesized from their respective iodocounterparts** 1** and** 2** by reaction with hexa-n-butylditin applying Pd as catalyst. Yields of** 3** and** 4** after purification using silica chromatography were moderate (ca. 40%).

Next, the stannylated precursors** 3** and** 4** were assessed in (“cold”) labelling tests. Here, the oxidative iodination conditions that are routinely applied in the radiolabelling of aryl-tin compounds, involving the use of [^123^I]-NaI in combination with H_2_O_2_, have been mimicked by replacing [^123^I]-NaI with regular (“cold”) NaI and by using this regular NaI at a low molar equivalent ratio of approximately 0.1 molequivalents relative to the aryl-tin precursor. These “cold” labelling tests have been executed to determine whether the precursors would produce the desired iodinated products, so product** 1** is from precursor** 3** and product** 2** is from precursor** 4**. As precursor** 3 **should give the required compound** 1 **in a single step, this precursor was preferred. However, after oxidative iodination of** 3** we did not find evidence for the formation of useful amounts of desired product** 1**, as monitored by HPLC-MS analysis. In contrast, we found, apart from the starting material** 3**, two product peaks with almost equal retention time where both peaks gave the same mass of* m/z* = 684. We assign these two peaks to the undesired byproducts** 5** and** 6** (see Scheme 1), where these two byproducts are formed by ortho iodination of the phenol ring. Note that the tri-n-butylstannyl groups in** 5** and** 6** are unaffected. Gratifyingly, oxidative iodination of precursor** 4** only gave the desired product** 2** with no trace of ortho iodination, again as monitored by HPLC-MS analysis.

According to the above “cold” labelling tests, the actual radiolabelling procedures have been executed using the MOM-protected precursor** 4**. In the first step, precursor** 4** was oxidatively iodinated using [^123^I]-NaI to arrive at the intermediate product [^123^I]-**2 **that was concentrated on an SPE C18 cartridge and thereafter collected in ethanol. In the second step H_2_SO_4_ was added to the ethanol solution to achieve deprotection of the MOM group. Heating of the reaction mixture was required to get efficient deprotection in several minutes. Finally, [^123^I]-**1 **was purified using a HPLC separation. As we have observed some side product formation in the MOM-deprotection step, we found a moderate overall radiochemical yield. Typically, the overall yield of the labelling process using precursor** 4** was 30–35% where the final radiochemical purity of [^123^I]-**1** exceeded 95%.

### 3.2. Ex Vivo Studies

**Figure 1:**
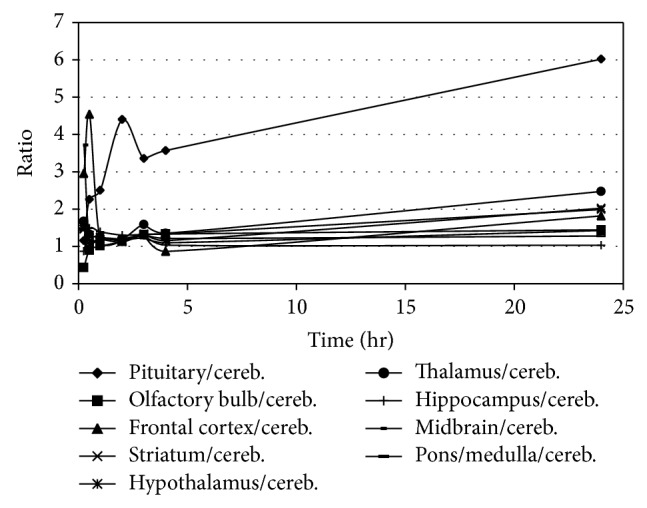
Results of biodistribution studies in Wistar rats. Uptake ratios of uptake in several brain areas compared to cerebellum uptake at different times after intravenous injection of [^123^I]-**1**.

The biodistribution studies showed that the uptake of [^123^I]-**1 **was higher in all brain areas than in blood from 15 min up to 1 h postinjection (p.i.) (Table 1 and Figure 1), suggesting efficient passage of the radiopharmaceutical through the blood-brain barrier. In almost all brain areas, [^123^I]-**1 **uptake was higher than in the cerebellum (Tables 1 and 2) from 15 min to 24 h p.i. As can be seen in Table 2 and Figure 1, the mean ratio of striatum/cerebellum binding was about 1.3 and was stable for the period of 30 min to 4 h p.i. In addition, the variation in binding was low in this period; therefore 2 h p.i. was chosen as time point to sacrifice the rats during the subsequent storage phosphor imaging experiment (see Tables 1 and 2 and Figure 1). Uptake in the pituitary, another part of the brain with a relatively high density of dopamine D_2_ receptor, was also relatively high.

Regarding the [^123^I]-**1** uptake in the peripheral organs and tissues, initially an intense uptake was seen in fat, muscle, heart, and spleen (15 min) followed by a washout (at 30 min and later, Table 1). However in lung tissue, thymus, and liver, uptake peaked at 30 min. In the heart and kidney, uptake peaked at 1 h. In the lung, liver, and kidney uptake remained more or less stable for the 1 h–4 h time period, with a second peak of uptake in the lung and kidney at 3 h and in the liver at 4 h. Also in blood a second peak was observed after 1 h p.i.

**Figure 2:**
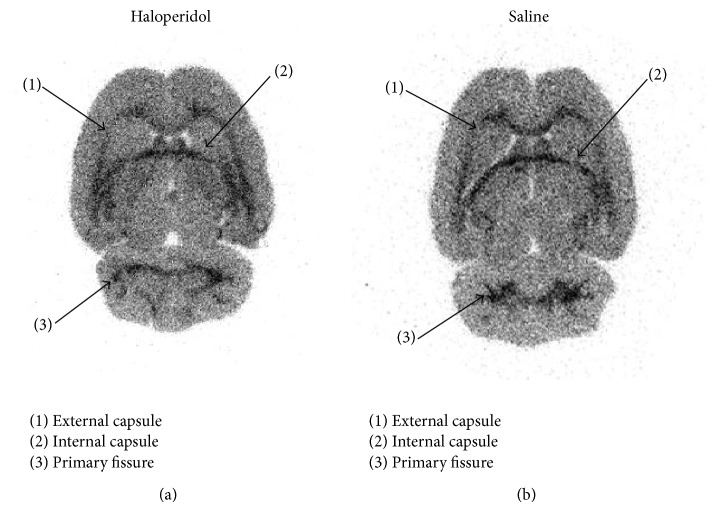
Ex vivo storage phosphor imaging slice experiment of [^123^I]-**1** (2 h p.i.). (a) This rat received 1 mg/kg haloperidol i.v. 5 min prior to injection of the radioligand. (b) Control rat received saline 5 min i.v. prior to injection.

In the storage phosphor imaging experiments, no specific striatal binding was observed for [^123^I]-**1** (Figure 2). Interestingly, high uptake in white matter was observed. In addition, no specific binding was visualized in other brain areas including the globus pallidus.

## 4. Discussion

Currently we are developing new agonist radiopharmaceuticals to visualize dopamine D_2/3_ receptors in their high-affinity state in the human brain for centers without an on-site cyclotron. Therefore a series of ligands were synthesized and tested in vitro [24]. On the basis of promising in vitro results, one of the assayed compounds (i.e., compound** 1**) was selected as a potential SPECT ligand. In this work we show the successful labelling of compound** 1** with iodine-123, as well as the ex vivo characterization in the rat.

Molecules** 3** and** 4** were prepared as potential precursors to produce [^123^I]-**1**, and “cold” labelling tests using NaI and oxidative conditions were conducted to assess the usefulness of these two precursors. For molecule** 3** undesired ortho iodination was shown to be preferred over iodination at the activated para-aryl-stannyl position. Indeed, the phenol ring in molecule** 3** is quite electron rich and is therefore prone to undergo ortho iodination. In contrast, the MOM-protected molecule** 4** only showed iodination at the activated para-aryl-stannyl position. Apparently, MOM-protection deactivates the phenol ring to such an extent that ortho iodination is prohibited and that iodination at the tri-n-butyltin site becomes favored and can occur exclusively.

**Scheme 2:**
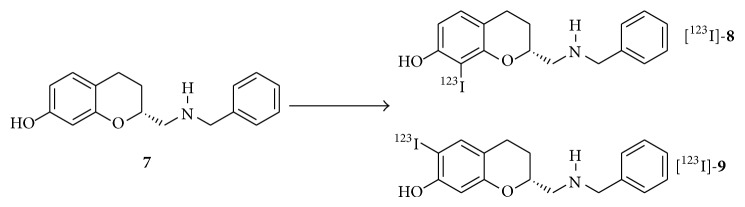
Hypothetical and prospective oxidative radiolabelling of AMC molecule** 7** to the potential tracers [^123^I]-**8** and [^123^I]-**9**. For** 8** and** 9**, labelling occurs at the 2- and 6-position of the phenol ring, respectively.

Although the oxidative ortho iodination of precursor** 3** was not desired for this current study, it can be considered for the preparation of the two prospective labelled compounds [^123^I]-**8** and [^123^I]-**9** (see Scheme 2) by using the simple precursor (R) N-[7-hydroxychroman-2-yl]-methyl benzyl amine (**7**). Molecule** 7** has previously been reported by Mewshaw and coworkers [25]; in the same work by Mewshaw et al. it has been shown that chlorination at the ortho 6-position of the phenol ring of AMC** 7** does affect the affinity for the D_2/3_ receptor somewhat, but the chlorinated species still displays a high-affinity and a high selectivity towards D_2/3_ receptor as compared to binding to other receptors. We have nevertheless not pursued the preparation of [^123^I]-**8** and [^123^I]-**9**, as we anticipate that the AMC pharmacophore will be affected by the introduction of the large iodogroup ortho to the hydroxy moiety. Furthermore, and as a more practically inclined notion, it may be difficult to effectively separate [^123^I]-**8** and [^123^I]-**9**, as both molecules will be formed in the iodination step and as** 8** and** 9** will have very similar chromatographic retention behavior.

The radiosynthesis of [^123^I]-**1** proceeded in two steps from precursor** 4**, giving a moderate radiochemical yield (30–35%) and a good radiochemical purity (>95%). We have so far not pursued further improvements of this radiolabelling process, as the acquired radiochemical purity was considered acceptable for the ex vivo testing of [^123^I]-**1**, while the radiochemical yield, although moderate, nevertheless permitted the preparation of sufficient amounts of radiolabelled compound for the execution of all planned tests.

In biodistribution studies, it was found that [^123^I]-**1 **had initially a higher uptake in the brain than in the blood suggesting rapid passage of the blood-brain barrier which is one of the crucial properties of a potential radiopharmaceutical for receptors in brain [29, 30].

Further biodistribution data showed that, in almost all areas of the brain, including the striatum, the [^123^I]-**1 **uptake was higher than in the cerebellum. However in the storage phosphor imaging experiments it appeared that no binding in the striatum was blocked by the dopamine receptor antagonist haloperidol, suggesting absence of specific displaceable binding to dopamine D_2/3_ receptor. The discrepancy between the biodistribution and storage phosphor imaging studies may be explained as follows. [^123^I]-**1** does bind in vivo to receptors that are expressed outside the cerebellum but are not blocked by haloperidol. Since haloperidol binds not only to D_2/3/4_ receptors but also to the sigma-1 sites [31] and *α*_1_-adrenoceptors [32], it is unlikely that [^123^I]-**1** binds to these receptors in vivo. However, further evaluations are needed to identify the receptor to which this novel radiotracer may bind. Nevertheless, the radiotracer may be too lipophilic to visualize D_2/3_ receptor in vivo, and consequently our findings do not support the start of further studies in humans.

In the storage phosphor imaging studies it was shown that [^123^I]-**1** binds to white matter, which suggests that the tracer has a relatively high lipophilicity. In line with this finding is the high uptake in peripheral fat tissue. This finding was unexpected since the calculated lipophilicity (log⁡⁡D) was within the proper range for developing a radiopharmaceutical [29, 30]. However, also in other studies the calculated lipophilicity did not always predict the success of a novel radiotracer [29] and calculated log⁡⁡D values can sometimes deviate from experimentally determined values [33]. In the biodistribution experiment a second peak was observed in the uptake of iodine-123 in blood at 1 h; this could be explained by a reabsorption in the duodenum of iodinated metabolites formed in the liver that are secreted in bile fluid (enterohepatic circulation). The fact that** 1** is excreted in bile and undergoes the hepatic circulation may also confirm that it has high fat solubility and thus a high lipophilicity.

Another hypothetical reason that we observed no specific binding of [^123^I]-**1** in the brain might be active efflux of [^123^I]-**1** through, for example, a PgP pump. However, we believe that this is unlikely since we recently observed that several other compounds of our AMCs showed to be no substrates for this efflux pump (unpublished data). Also, we cannot exclude the fact that radiolabelled metabolites were formed that have passed the blood-brain barrier. However, for a similar compound (AMC20), we found that 95% of the radioactivity from a brain tissue extract (35 min after injection) was intact ^18^F-AMC20 and that radiometabolites of ^18^F-AMC20 were hydrophilic and did not pass the blood-brain barrier [34]. Since our present compound has a similar structure, we believe that it is unlikely that formation of radiometabolites will have significantly influenced the results.

The in vitro experiments showed that the compound** 1 **binds with high-affinity to dopamine D_4_ receptor. However, the storage phosphor imaging studies also showed no specific binding in the D_4_-rich globus pallidus. Given that this tracer did not bind in vivo to the other D_2_ receptor-like receptors (i.e., D_2/3_ receptor), it would have been unexpected that this tracer binds selectively to the D_4_ receptor in vivo. However, only future blocking studies using selective dopamine D_4_ receptor agents can prove this postulate.

In our previous study [24] where we explored the new group of potential agonist tracers on in vitro properties, two of the most promising fluorine containing compounds were fluorine-18 labelled successfully. Both displayed specific binding to dopamine D_2/3_ receptors in vitro in rat brain slices in autoradiography experiments, which encourages further in vivo evaluations, including storage phosphor imaging.

## 5. Conclusion

We have successfully prepared the radiolabelled D_2/3_ receptor agonist [^123^I]-**1**. Although this novel radiotracer specifically binds to D_2/3_ receptors in vitro, its signal-to-noise ratios in rodents were low and no specific binding was observed which prevent further use in humans. Particularly, the performed biodistribution studies suggested higher binding in the D_2/3_ receptor-rich striatum than in brain areas devoid of D_2/3_ receptors, while the final storage phosphor imaging studies showed no selective binding of [^123^I]-**1** to central D_2/3_ receptors in rats. Still, this new scaffold may be a good reference and starting point for further studies aiming at the development of in-demand D_2/3_ receptor agonist SPECT tracers suitable for in vivo application in humans.

## Figures and Tables

**Table 1:** Biodistribution of ^123^I radioactivity (%ID × kg/g) at different times after intravenous injection of [^123^I]-**1** in male rats (*n* = 4 per group). Data represent mean ± SD.

	15 min	30 min	1 h	2 h	3 h	4 h	24 h
Blood	0.291 ± 0.190	0.486 ± 0.291	0.557 ± 0.087	0.446 ± 0.105	0.428 ± 0.082	0.393 ± 0.136	0.081 ± 0.049
Fat	3.080 ± 2.656	0.674 ± 0.102	0.509 ± 0.024	0.365 ± 0.094	0.337 ± 0.065	0.174 ± 0.056	0.016 ± 0.005
Muscle	0.412 ± 0.466	0.363 ± 0.049	0.244 ± 0.023	0.124 ± 0.028	0.119 ± 0.028	0.084 ± 0.041	0.012 ± 0.004
Thymus	0.365 ± 0.507	1.776 ± 0.200	1.591 ± 0.169	1.083 ± 0.454	0.946 ± 0.150	0.815 ± 0.244	0.024 ± 0.001
Heart	1.678 ± 1.142	0.658 ± 0.141	0.962 ± 1.043	0.669 ± 0.348	0.372 ± 0.265	0.180 ± 0.044	0.033 ± 0.005
Lung	3.483 ± 3.534	5.673 ± 2.526	1.622 ± 0.848	0.390 ± 0.302	0.936 ± 0.762	0.573 ± 0.307	0.040 ± 0.005
Liver	1.316 ± 0.773	2.644 ± 0.361	2.470 ± 0.449	2.713 ± 0.487	2.387 ± 1.400	3.164 ± 0.824	0.260 ± 0.064
Spleen	2.428 ± 1.971	1.270 ± 0.282	0.749 ± 0.093	0.266 ± 0.047	0.284 ± 0.092	0.166 ± 0.087	0.022 ± 0.003
Kidney	1.618 ± 0.774	1.735 ± 0.353	1.770 ± 0.302	1.081 ± 0.154	1.336 ± 0.175	1.005 ± 0.397	0.150 ± 0.026
Pituitary	1.401^*^	3.367 ± 1.033	1.561 ± 0.538	0.918 ± 0.632	0.548 ± 0.149	0.307 ± 0.056	0.082 ± 0.006
Olfactory b.	0.445 ± 0.439	1.325 ± 0.402	0.631 ± 0.036	0.218 ± 0.025	0.234 ± 0.087	0.112 ± 0.043	0.020 ± 0.001
Frontal cortex	2.567 ± 1.933	2.329 ± 0.904	0.722 ± 0.061	0.223 ± 0.028	0.232 ± 0.095	0.109 ± 0.057	0.024 ± 0.002
Striatum	1.231 ± 1.299	1.947 ± 0.666	0.779 ± 0.037	0.226 ± 0.034	0.232 ± 0.065	0.101 ± 0.051	0.027 ± 0.010
Hypothalamus	1.335 ± 0.766	1.699 ± 0.526	0.747 ± 0.111	0.218 ± 0.036	0.233 ± 0.070	0.114 ± 0.051	0.027 ± 0.005
Thalamus	1.556 ± 1.015	1.978 ± 0.618	0.779 ± 0.049	0.234 ± 0.037	0.279 ± 0.091	0.114 ± 0.047	0.033 ± 0.006
Hippocampus	0.826 ± 0.475	2.155 ± 0.675	0.866 ± 0.089	0.255 ± 0.040	0.230 ± 0.085	0.093 ± 0.059	0.014 ± 0.001
Midbrain	3.566 ± 4.660	1.715 ± 0.577	0.754 ± 0.040	0.229 ± 0.046	0.214 ± 0.062	0.098 ± 0.059	0.020 ± 0.003
Pons/medulla	1.188 ± 0.587	1.486 ± 0.274	0.761 ± 0.040	0.237 ± 0.045	0.229 ± 0.066	0.106 ± 0.057	0.017 ± 0.001
Cerebellum	0.858 ± 0.384	1.496 ± 0.478	0.627 ± 0.035	0.196 ± 0.037	0.178 ± 0.061	0.087 ± 0.043	0.014 ± 0.002

^*^At this time point and in this brain area, only 1 sample was available.

**Table 2:** Brain areas to cerebellum uptake ratios at different times after intravenous injection of [^123^I]-**1**.

	15 min	30 min	1 h	2 h	3 h	4 h	24 h
Pituitary/cereb.	1.157^*^	2.262 ± 0.233	2.502 ± 0.883	4.399 ± 2.113	3.352 ± 1.490	3.570 ± 2.544	6.021 ± 0.577
Olfactory b./cereb.	0.431 ± 0.330	0.893 ± 0.067	1.007 ± 0.050	1.125 ± 0.089	1.314 ± 0.150	1.333 ± 0.124	1.444 ± 0.163
Frontal cortex/cereb.	2.962 ± 1.817	1.543 ± 0.142	1.154 ± 0.095	1.145 ± 0.079	1.315 ± 0.360	0.865 ± 0.580	1.818 ± 0.372
Striatum/cereb.	1.455 ± 1.265	1.294 ± 0.061	1.246 ± 0.103	1.155 ± 0.069	1.331 ± 0.126	1.156 ± 0.080	2.023 ± 0.872
Hypothalamus/cereb.	1.512 ± 0.546	1.139 ± 0.014	1.189 ± 0.132	1.116 ± 0.085	1.326 ± 0.053	1.332 ± 0.103	1.989 ± 0.574
Thalamus/cereb.	1.670 ± 0.908	1.325 ± 0.074	1.243 ± 0.042	1.200 ± 0.067	1.582 ± 0.101	1.351 ± 0.175	2.473 ± 0.599
Hippocampus/cereb.	0.870 ± 0.303	1.479 ± 0.466	1.390 ± 0.210	1.301 ± 0.047	1.294 ± 0.112	1.035 ± 0.114	1.031 ± 0.090
Midbrain/cereb.	3.712 ± 4.715	1.144 ± 0.051	1.207 ± 0.109	1.168 ± 0.080	1.224 ± 0.090	1.097 ± 0.086	1.426 ± 0.204
Pons/medulla/cereb.	1.470 ± 0.520	1.036 ± 0.212	1.218 ± 0.109	1.205 ± 0.016	1.310 ± 0.114	1.214 ± 0.133	1.278 ± 0.211

^*^At this time point and in this brain area, only 1 sample was available.

## Abbreviations

AMC: 2-Aminomethylchroman
moleqs: Molar equivalents
MOM: Methoxymethoxy
PDA: Photodiode array
TOH: At the time of handling.
